# Cognitive-Based Interventions Break Gender Stereotypes in Kindergarten Children

**DOI:** 10.3390/ijerph182413052

**Published:** 2021-12-10

**Authors:** Yi Chung, Hsin-Hui Huang

**Affiliations:** 1 College of Human Development and Health, National Taipei University of Nursing and Health Sciences, Taipei 112303, Taiwan; m9306009@gmail.com; 2 Department of Infant and Child Care, National Taipei University of Nursing and Health Sciences, Taipei 112303, Taiwan

**Keywords:** gender stereotype, cross-classification, gender equality, multiple conceptual systems, children

## Abstract

Despite the growing recognition of gender equality worldwide, plausible strategies that reduce young children’s gender stereotypes remain limited. Cognitive-based interventions have been widely used in school settings and have been suggested to play important roles in children’s gender stereotyping and in their processing of counter-stereotypic information. We aimed to determine whether exposure to counter-stereotypical information could break gender stereotypes in kindergarten children. Fifty-four children (61–79 months old) from two public kindergarten classes in northern Taiwan participated in this study. One of the two classes was randomly selected as the experimental group (*n* = 28), and the other was the control group (*n* = 26). The experimental group consisted of a gender equality curriculum including script relationship training for two months, while the control group continued their regular curriculum. The picture classification task (PCT) was measured before and after the intervention to assess gender stereotypes. Before interventions, 87.50% of the children chose a gender stereotypic relationship, while 12.50% chose script/other relationships in PCT. After the interventions, the gender stereotypic relationship dropped to 73.22% in the experimental group. Children in the control group were more likely to maintain their gender stereotypic relationship choices in PCTs. Our findings suggest that cognitive-based interventions, such as a gender equality curriculum, have the potential to break gender stereotypes in kindergarten children.

## 1. Introduction

### 1.1. Development of Gender Stereotype

Humans have a natural tendency to classify things. Human beings possess necessary classification abilities from infancy, which develops gradually with cognitive ability and learning experience [1]. Children form concepts by classifying things according to certain similarities, which is the basis for them to understand and organize the world [2]. Grouping according to the essential traits of individuals is called social categorization. Human beings often categorize individuals based on background characteristics (e.g., gender, age, race, or religion), personality traits, interests (e.g., extroversion and hobbies), and occupation [3] and apply their presupposition of the group to individuals.

Social categorization involves two processes. One is to emphasize the characteristics shared within a group and to reduce individual differences among group members, thus generating stereotypes; the other is to strengthen and concentrate on the differences among different groups [3]. Social categorization can help individuals organize social and interpersonal information efficiently. However, if lacking flexibility, social categorization becomes an obstacle for individuals to understand the world.

Gender is one of the most important social categories. Infants and young children quickly develop gender-related concepts that lead them to engage in specific activities, to discover exciting things, and to achieve goals in the process [4]. Studies show that infants aged 3 to 4 months can distinguish between male and female groups by facial features alone [5]. By about 9 to 11 months old, they can distinguish between male and female groups by face and have the ability to intermodally associate features (e.g., associating faces and voices) [6]. Many young children before two years of age can integrate more clues (e.g., hairstyle, voice, and clothing) to correctly label men or women (including themselves), to verbally express gender nouns (e.g., girl and boy), and to understand gender differences [7,8,9]. When they see pictures of males engaging in female stereotypic activities (e.g., applying lipstick), they stare at the pictures for a longer time [10]. Girls know about gender earlier than boys, and 2-year-old girls can associate toys (e.g., dolls or cars) with gender [11].

Children continuously learn about the differences between male and female behaviors and objects of use in a specific culture through observation. Around the age of three, children learn about gender stability and show gender stereotyping similar to adults regarding toys, clothes, activities, games, colors, and even specific personality descriptions [12]. By around five years old, they begin to develop gender identity and to possess gender stereotyping of personal–social attributes [13]. At this age, children tend to think that they are more similar to their same-gender peers and are more likely to associate themselves with characteristics that fit the gender stereotype [14]. After entering primary school, children’s gender stereotyping extends to more dimensions, such as career choices, sports, motives to learn subjects, and ambition [15], which has a long-term impact on the cognition and behaviors of individuals. 

From an ecological perspective, young children’s socialization of gender stereotypes comes from their family and school and from more distant systems, including cultural values [16]. Nevertheless, gender stereotypes seem to be a universal phenomenon and to develop from an early age. Although most studies mentioned focused on children in North American, children in other cultures such as Spain [16], Austria [17], and Taiwan [18,19] seem to share similar beliefs and attitudes toward gender roles. 

To measure the gender stereotyping of children, Edelbrock and Sugawara [20] developed the Sex-Role Learning Index (SERLI), in which children are asked to categorize pictures of gender stereotypic activities as “boys”, “girls”, or “both boys and girls”. In recent years, Banse et al. [21] developed the Active Interference Paradigm (AIP) suitable for young children, in which young children are asked to assign toys to boys and girls in either a stereotype-congruent or a stereotype-incongruent manner as quickly as possible. The accuracy rate of stereotype-congruent classifications indicates gender stereotypic knowledge while the accuracy rate of stereotype-incongruent classifications indicates gender stereotype flexibility. The research shows that 3-year-old children generally have gender stereotypic knowledge, which is strengthened year by year until about five years old when their gender stereotypic knowledge remains stable at high levels [21,22]. On the other hand, gender stereotype flexibility declines year by year from three years old, which rebounds only from five years old, and then reaches the ceiling effect by the ages of ten to twelve [21,23,24]. One research investigating the factors associated with gender stereotyping found that preschool girls’ gender stereotypic knowledge is higher than that of boys, but there are no gender differences in gender stereotype flexibility [23]. Additionally, the stereotype of men’s roles is more rigid than that of women [25]. 

Overall, preschool children already have stable concepts of gender, and these become essential prior knowledge. From this prior knowledge, our brains naturally collect and extract relevant information to make classification learning faster and easier when responding to a stimulus [26]. However, the system’s main disadvantage is that it prevents children from seeing or imagining other potentials beyond their prior knowledge of gender. Fortunately, children’s views on gender are not unshakable. Citing from Devine’s dissociation model of prejudice and stereotyping, Banse et al. [21] suggested that cognitive effort and control of the process would weaken gender stereotypic knowledge. Following the view of cognitive flexibility, connectionist models propose that updating the weights for connections between gender-related concepts may enhance children’s gender flexibility [27]. 

### 1.2. Cognitive Basis of Children’s Classification

Most previous studies have used picture classification tasks (PCT) to investigate whether children’s gender conceptual schema changed. The premise of such test tools is that children have multiple conceptual systems such as adults [28]. Common conceptual systems include the taxonomic system, thematic or complementary system, and script system. A taxonomic system includes subordinate, basic, and superordinate categories (e.g., curly haired dog–dog–mammal) [29]. A thematic or complementary system refers to associating objects according to their functions in classification, as they often appear together (e.g., cats and fish), and a script system regards the different things that may appear in daily recurring events as one category, such as including cereal and bread in the breakfast script [29]. However, objects with a script relation may not necessarily coexist in the same space or contact each other, as cereal and bread are not necessarily served simultaneously.

Evidence suggests that even a one-year-old infant makes classifications based on the taxonomic system [30]. Children over two years of age have cross-classification abilities, meaning they classify the same item into more than one category [29]. Nguyen and colleagues conducted a series of studies to confirm that preschool children can use taxonomic and script systems to classify items from several domains [29,31]. Similarly, Smiley and Brown [32] showed that, even though children make classification using a thematic system, they could still explain the taxonomic relationship between objects if asked about it.

Past studies have demonstrated that children can generate new classification concepts inconsistent with their prior knowledge through observation and cognitive training [33,34]. Therefore, appropriate cognitive training or learning experiences of non-traditional gender roles is likely to provide new contexts for judgment, to activate atypical gender representations, and to make new connections of the underlying conceptual network. For example, exposure to gender-atypical storybooks increases children’s play with gender-atypical toys and relaxes their judgments about gendered activities and occupations [35].

### 1.3. Motivation

The first social environment that children experience is their family, which is also the most influential environment for children, where parents play an extremely important role. Psychoanalysts believe that young children eventually identify with the same-gender parents and learn their behavior; social learning theorists think that parents are one of the models for children’s behavioral imitation learning; cognitivists believe that young children learn to behave similar to a same-gender adult [19]. The so-called “gender role” is often regarded as a “gender stereotype”, which refers to the recognized status, behavior, attitude, and expectations of men and women in society, such as the general public thinking that raising children is a woman’s duty and that men should bear the burden of household expenses [36]. The influence of parents on the gender role of their children can be summarized in two main aspects. One is the various disciplines directly given to children by parents, including the permission and prohibition of behavior, the formulation of internal family rules, and the provision of various resources. On the other hand, children develop adaptive behaviors by observing and imitating the characteristics or behaviors of their parents. Behavior, which is usually an imperceptible process, means that children gradually accept the parents’ gender values within their interactions [36,37]. 

Even if parents support children playing with opposite-gender toys, children still believe that parents actually support them playing with toys of their own gender [38], and they also noted that the parent–child interactions reflect the adult values and beliefs, as children try to understand their parents’ expectations and limitations. Parents’ gender perspectives are mainly presented by the way they treat their children. Parents often treat babies of different genders differently based on their own gender awareness, with especially mothers being often less aware of their different treatment methods [39]. Mothers with more traditional gender role attitudes may enhance the typical social behaviors of their children. Specifically, the difference between parents’ treatment of children of different genders is reflected in the parents’ response to children’s behavior, the choice of toys, and the distribution of housework [19]. If the child is a boy, the parents encourage large body movements and provide more physical stimulation and the parents have a more positive view of the boy’s physical activity [40]. Parents not only encourage children to perform appropriate gender role behaviors but also punish inappropriate behaviors, such as girls playing with dolls, dancing, and dressing and boys playing with blocks are enhanced, while girls running, jumping, or climbing and boys playing with dolls are scolded [19]. In addition, economic differences between genders as well as parents’ jobs possibly influence stereotypes toward a differentiation in the roles for managing a household, such as women being restricted and limited to certain roles and sometimes experiencing physical and psychological violence. Overall, the influence of parents on the concept of children’s gender can be described as profound and enormous. In the process of socialization, the main factors affecting the formation of children’s gender roles are family, school, peers, mass media, etc. [41]. Therefore, we are motivated to investigate whether school interventions can break gender stereotypes in children.

### 1.4. The Present Study

Past research about young children’s ideas of gender focused more on the development, influential factors, and effects of gender stereotyping. Less research aimed to improve young children’s gender stereotype flexibility, let alone interventions based on children’s cognitive characteristics. According to the review by Lenton et al. [27], most research on automatic gender stereotypes has been conducted with young adults. They concluded three intervention categories, including attentional distraction, the salience of within-category heterogeneity, and stereotype suppression. The second category might be a better approach among the three because it would activate counter-stereotypical subtypes [27]. In light of the above, gender equality curriculum interventions for kindergarten children were developed. The interventions provide script relationship training by referring to the cross-classification that children possess and aims to rebuild children’s belief in the concept of gender through picture-book stories and discussions. 

The current study investigated the effects of cognitive training on loosening young children’s gender stereotyping and the differential role of children’s background variables. The plan for analysis was to first present quantitative data on children’s classification choices after the intervention and then to supplement it with qualitative data from children’s discourse to provide the context of their cognitive rationale. 

## 2. Materials and Methods

### 2.1. Participants

From two public kindergarten classes, 54 children (30 boys and 24 girls) in northern Taiwan participated in this study. One of the two classes was randomly selected as the experimental group (*n* = 28), and the other was the control group (*n* = 26). The average age of the children was 72.39 months (61–79 months). All households had two parents. A total of 33 fathers (61.10%) and 24 mothers (44.44%) had a degree above junior college. A total of 28 fathers (51.9%) and 16 mothers (29.6%) were white-collar professionals, and 26 fathers (48.1%) and 38 mothers (70.4%) were blue-collar workers.

### 2.2. Measures

#### 2.2.1. Assessment: Picture Classification Task (PCT)

The classification materials included 8 sets of pictures, with 3 pictures in each set, totaling 24 pictures. The pictures were compiled based on past literature [20,34], as shown in Table 1. In each set of pictures, the first was the target picture (e.g., a boy); the second was the stimulus picture A (e.g., a robot), with the content being consistent with the gender stereotype of the target picture; and the third was the stimulus picture B (e.g., a doll), with the content being inconsistent with the gender stereotype of the target picture. There was a script relationship between two stimulus pictures that aligns with the kindergarten children’s experiences. Masculine and feminine content accounted for half of the target pictures. 

**Figure 1:**
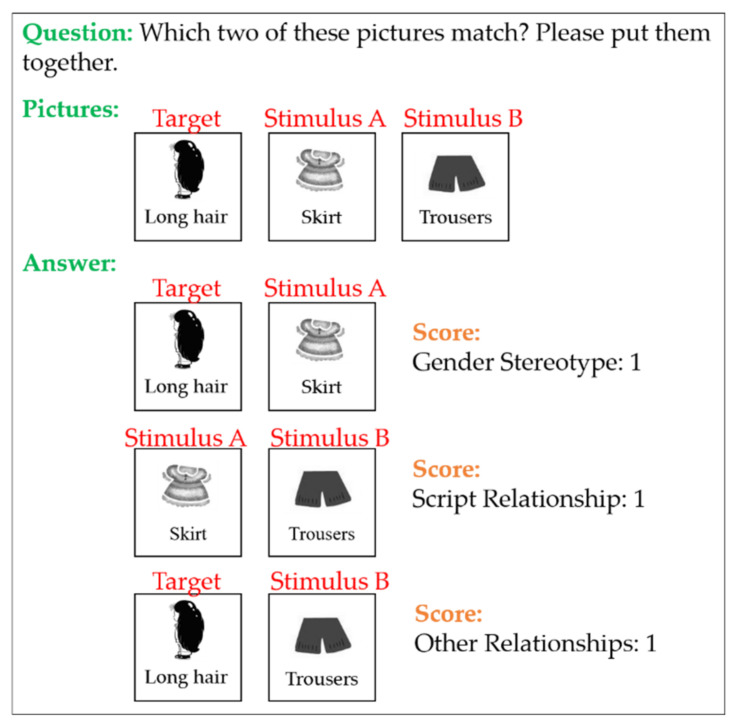
The scoring protocol of the picture classification task. Matching the target and stimulus A picture scores 1 point in a “gender stereotypic relationship”; matching stimulus A and B scores 1 point in a “script relationship”; and matching target and stimulus B scores 1 point in “other relationships”.

The PCT adopted a one-on-one interview method. The order of each picture set was randomly arranged, and each set was randomly placed on the desktop to reduce the position effect. The testing procedure included putting one picture set on the desktop, asking the children to confirm their contents one by one, and then asking, *“Which two of these pictures match? Please put them together”* while emphasizing that there is no right or wrong answer to the question. After the children chose the pictures, the interviewer asked again, *“Why do you want to put these two pictures together?”* and video-recorded the children’s answers (Figure 1). 

The scoring method was as follows: matching target pictures and stimulus picture A scored 1 point in a “gender stereotypic relationship”; matching both stimulus pictures A and B scored 1 point in a “script relationship”; and matching target pictures and stimulus picture B scored 1 point in “other relationships”. The total score of the PCT was 8 points. The children’s answers were transcribed verbatim.

In the present study, a variation in PCT was developed to test the children’s automatic gender stereotypes in a class group before interventions. The PCT worksheet contained eight sets of stimulus pictures A and B, but the positions of A and B in each set were randomly interlaced. The modified protocols were similar to the PCT. First, the assessor showed the target pictures (such as “long hair”) one by one and asked, “What is this?” to ensure that the children understood the content of the pictures. If the children gave wrong answers or no answer, the assessor gave them the correct answers. The children were then asked, *“What do you think long hair should be matched with?”* and gave their answers by circling pictures A or B on the worksheet. The assessor emphasized that only one of the two answers should be selected and that there are no right or wrong answers.

The worksheets were then collected to calculate the scores. Circling stimulus picture A received 1 point while circling stimulus picture B received 0 points. The scores of each child ranged from 0 to 8, and the higher the score, the more stable the children’s gender stereotyping. The purpose of the task was to assure the children were in stable gender stereotyping before intervention. The PCT was assessed before (pretest) and after (posttest) the intervention. One week after the pretest, the 8-week intervention curriculum was implemented in the experimental group.

#### 2.2.2. Intervention: Gender Equality Curriculum (GEC)

Eight picture books on the theme of gender equality suitable for kindergarten children were selected, including *Oliver Button is a sissy*; *The paper bag princess*; *William’s Doll*; *Fox hatches an egg*; *Rawr! You look delicious*; *Where’s our mama?*; *Piggybook*; and *Sara wants to join the circus*. All books were trade books of high literary quality and covered various gender issues, including gender roles, gender bias, gender stereotypes, etc. We developed 16 lesson plans according to the picture books. The teacher read one picture book and engaged the children in discussions and extended activities for 30–40 min each session, two times per week. For example, *William’s Doll* was the book chosen for week 3. The story follows William, a young boy who wishes for a doll to care for. William’s father is unhappy with his request, instead giving William toys that he considers to be more gender appropriate. Finally, William’s grandmother fulfills William’s request, explaining to William’s father that the doll allows for William to practice good parenting [42]. In the first session, the teacher asked the children to bring one of their favorite toys to share in class and then discussed questions, such as why they liked their toys, any differences between the toys brought by boys and girls, whose toys would they like to play and why, and whether they would share their toys with others and why. In the second session, the teacher read *William’s Doll* and asked more questions about why people did not like the same thing; how would they feel when others did not like what they liked; when others mocked what they liked; and what would they do if they were William’s brother, classmates, or father. During the discussions, the teacher asked open questions and encouraged children to share their experiences, to reflect, and to express opinions. The intervention lasted for eight weeks, and all conversations and activities were video-recorded. The researcher led the experimental class to carry out the eight-week curriculum on gender equality, while the control group carried out their regular curriculum. The PCT was conducted one week after the intervention.

## 3. Results

### 3.1. The Pretest PCT

To ensure that the internal validity was not threatened by selection bias, chi-square tests were used to compare background variables between the experimental and the control group. There was no difference between the groups in the children’s gender (*χ^2^* = 0.06, *df* = 1, *p* = 0.808), father’s educational background (*χ*^2^ = 0.39, *df* = 1, *p* = 0.535), mother’s educational background (*χ*^2^ = 0.09, *df* = 1, *p* = 0.761), father’s job (*χ*^2^ = 0.08, *df* = 1, *p* = 0.777), and mother’s job (*χ*^2^ = 0.18, *df* = 1, *p* = 0.675). Homogeneity testing also showed no significant difference between the groups before intervention (*χ*^2^ = 0.34, *df* = 2, *p* = 0.844) (Table 2).

In the pretest PCT, there were 432 paired choices made by the children, of which 378 were the gender stereotypic relationship (87.50%), 36 were the script relationship (8.30%), and 18 were other relationships (4.20%), as shown in Table 3. The three types of classification choices did not appear random according to the goodness-of-fit test. All children scored more than 7 points out of 8, indicating stable gender stereotyping.

The reasons why the children choose the gender stereotypic relationship mostly echo the thematic system; that is, the association was formed because gender and an object often appear together or because gender and an ability trait are often mentioned at the same time. Further analysis showed that there were two subtypes of children’s responses. The first was to emphasize that one gender is more suitable for or likes a particular object, or is more capable of doing something, such as “long hair goes better with skirts,” “fathers are better at fixing things,” and “girls all like to keep things neat.” These responses reflected the children’s stereotypic knowledge about genders. The second was to connect a gender with particular objects or abilities, such as “this is his (father’s) hammer,” “soldiers need to drive for fighting,” “girls’ clothes have bows,” and “dads cannot feed milk to babies.” These answers show that children have a clear demarcation of gender and that their views are rigid and inflexible.

Although most children chose the gender stereotypic relationship, a few children choose the script relationship. The reasons for choosing the script relationship were not limited to the script system discussed in training but also included the taxonomic and thematic systems. For example, children answered that “these two (toy car and toy kitchen) are toys,” “skirts are for girls, but girls can also wear trousers, because skirts and trousers are the same kinds,” and “they are both made of cloth (bow tie and tie).” The above responses demonstrate that children categorize the relationship between two objects at the same level or even give it a name explicitly, representing their understanding of the taxonomic system. Examples of responses with the thematic system include, “parents will have babies after getting married because they (father and mother) can chat with each other,” that is, the association is formed according to characteristics that appear together. Examples of responses with the script system include, “you should wear skirts and trousers when you go out, or others will see you (naked),” “both (razor and lipstick) are available in department stores,” and “because both (brooms and heavy box) are needed when we have to work and sweep the floor,” meaning they regard different objects that appear in recurring events as being of the same category. The above supports that children have multiple conceptual systems. 

#### Impact of Background Factors

Homogeneity testing was used to investigate the distribution of gender in children’s classification choices, and the results were shown in Table 3. Boys choosing the script relationship showed a significantly higher proportion than girls (*χ*^2^ = 11.31, *df* = 2, *p* = 0.004).

The Pearson product–moment correlation was used to analyze the relationship between children’s age in months and the counts of the three classification choices. Children’s age was not correlated with the gender stereotypic relationship (*r* = −0.06, *p* = 0.656), script relationship (*r* = 0.19, *p* = 0.174), and other relationships (*r* = −0.24, *p* = 0.083).

### 3.2. Intervention

During the eight weeks of intervention, children’s discourse about gender was influenced by picture books, as depicted in the excerpts below.

*William’s Doll*

Teacher (T): Do you think William can get the doll?

Student Shu (Shu): Yes, because he likes it very much.

T: How would you feel if everyone laughed at you and call you sissy?

Student Fu (Fu): Terrible.

Student Yu (Yu): Sad.

Student Ting (Ting): Lonely.

Student Yen (Yen): Unhappy.

T: So, do you think toys should be divided into girl toys and boy toys?

Class: No.

Student Lin (Lin): No, no need! Robots are not necessarily for boys, and dolls are not necessarily for girls.

Student Li (Li): No, it does not matter.

*Piggybook*

T: Would you be angry if you were Mrs. Piggott? What would you do? What should boys do?

Shu: Boys should help!

Lin: (Mrs. Piggott) Do not do anything! (Angry)

Ting: I’ll be angry, but I would not run away from home.

Student Chun: Boys should help with housework.

Student Ching: Boys should practice doing housework.

Fu: Boys will become pigs if they don’t do housework!

During the intervention, children in the experimental group, regardless of gender, could empathize with the protagonist’s problems and troubles in the picture book. Additionally, they expressed flexible opinions about the appearance, personality traits, toys, occupation, and housework division of labor for specific genders, which was quite different from the gender stereotypic thoughts in the pretest.

### 3.3. The Posttest PCT

Although the gender stereotypic relationship was still the most common choice, the percentage in the experimental group was lower than from the pretest (from 87.5% to 73.21%) in Table 4. The test of homogeneity was significant, suggesting differences in classification choices between groups (*χ*^2^ = 45.67, *df* = 2, *p <* 0.001). Compared with children in the control group, children in the experimental group were less likely to choose the gender stereotypic relationship but more likely to choose the script and other relationships.

To understand whether the change in children’s classification choice is related to the group or other background factors, we re-coded each question according to the choices before (pretest) and after (posttest) intervention, with the gender stereotypic relationship as A, the script relationship as B, and other relationships as C. If A in pretest and B in posttest were chosen, then the code was AB. In this way, nine new variables (AA, AB, AC, BA, BB, BC, CA, CB, and CC) were generated. We also examined the correlations with background variables such as parents’ educational level and job because they might be factors to control for in the regression analysis. The results showed that there were no significant differences in mother’s educational level and parents’ job. However, in father’s educational level, when binary logistic regression was used to analyze the nine new variables’ predictive effect with father’s educational level (0 = below junior college, 1 = above junior college) as the dependent variable, AA reached a significant level, *B* = 0.405, *Wald*(1) = 7.764, *p* = 0.005, odds ratio (*OR*) = 1.499. In other words, for children who chose the gender stereotypic relationship both before and after the study, his/her father’s educational level was 1.499 times more likely to be above junior college. When father’s educational level was controlled for the groups (0 = experimental group, 1 = control group) as the dependent variable, only AA reached a significant level, *B* = 0.438, *Wald*(1) = 5.124, *p* = 0.024, odds ratio (*OR*) = 1.55. That is to say, children who chose the gender stereotypic relationship are 1.55 times more likely to be from the control group and had less chance to alter his/her own stereotype patterns.

Classification of the gender stereotypic relationship. Of the 208 paired choices made by the children in the control group, 96.15% were the gender stereotypic relationship, which was higher than that of children in both groups (87.5%) before intervention. 

The reasons given by the children in the control group reflected gender stereotypic knowledge, such as “girls are suitable for sweeping the floor and boys like to play King Kong.” Only eight paired choices were not of the gender stereotypic relationship. Six out of these eight responses were from the same girl, whose responses showed that she remembered the script relationship before intervention, such as “both (toy car and toy kitchen) can be found in toy stores,” and “they (brooms and heavy box) are all sweeping supplies.” 

Compared with children in the control group, those in the experimental group chose the gender stereotypic relationship (73.21%) for more diversified reasons, such as explaining the relationship between gender and objects with narrations rich in plot:

The girl’s mum left home, then her dad went to work, and her brother went to school, so only she was left at home. Then, her brother told her to stay at home and take care of the house. However, the house was messy. He asked her to clean it, and then she did. (Girl and Broom)

Alternatively, they may admit that both genders can be associated with the object, showing better gender stereotype flexibility, but choose a gender stereotypic relationship because a gender is more commonly associated with the object. For example, one girl paired Boy and Robot and told the assessor, “Because boys can also play with dolls, but boys more often play with robots.”

Classification of the script and other relationships. Of the 224 responses given by children in the experimental group, 60 were not the gender stereotypic relationship, accounting for about 1/4. Some children quoted the discussion content in classroom activities to explain their classification reasons.

Because in The Paper Bag Princess, she... She found the paper bag that was not burned by the fire dragon. Then, she put it on. However, the prince did not like her. She still went home happily. (T: Why?) Because some people can also use (pointing to tie), like you did last time, right? (Doll + Tie)

The GEC intervention showed that at least 10 out of 28 children in the experimental group changed their perspectives about genders. The following are the reasons stated by several children on the same task item before and after the second session of the intervention.

Girls like to sweep the floor. (Girl + Brooms)

In that... Uh... because sometimes the girl will use that (heavy box) to carry things. (Girl + Heavy Box)

Mothers give birth to babies. Because fathers are at work, and mothers are on vacation so that they can stay with their babies. (Baby + Mother)

Dad does not have to go to the company; he can also... sometimes spend time with the baby. (Baby + Father)

Boys love playing with toy robots. (Boy+ Robot)

Because you have talked about William’s Doll, and then it is okay for a little boy to want a doll! (Boy + Doll)

From the children’s responses, we can see that some children’s gender stereotyping was changed after intervention, indicating that they started to have more flexible views on the appearance and preferences of a particular gender. For example, “girls do not necessarily need to wear skirts, they can also wear trousers,” “boys can also play with dolls,” or “girls also look beautiful with neckties.” The intervention had the same effect on changing the stereotype of the division of labor between males and females, for example, “mom can also work,” “dad can also take care of the baby,” or “because mom can also help with (hammer).” They believe that men and women should share housework. 

#### Impact of Background Factors

We used a binary logistic regression to examine the relation between AA and the children’s genders (0 = female and 1 = male) in the experimental group. The results showed that there was no correlation between the children’s genders and their classification choices. A Pearson product–moment correlation analysis of the relationship between children’s age in months and their classification choice of AA showed that there was a negative but not significant correlation (*r* = −0.345, *p* = 0.072).

## 4. Discussion

### 4.1. Effects of Interventions 

We examined the effects of cognitive-based interventions, GEC, on loosening children’s gender stereotyping. The results show that, after the GEC intervention, the gender stereotypic relationship was chosen less although it was still the primary classification choice of the children, which shows that gender stereotyping is a vital classification basis for preschool children. 

As Banse et al. concluded, individuals gradually shape their views on genders, and corresponding abilities and characteristics through observation and socialization from birth [21]. Therefore, gender stereotypic knowledge learned until preschool age becomes automatic and relatively resistant to change. Nevertheless, a GEC intervention still has a small but significant effect on loosening children’s gender stereotypes. The script relationship training designed in this study aimed to make use of children’s cross-classification ability to instruct the children to understand the script relationship between two stimulus pictures. That is, the training was intended to accentuate another way to classify the pictures instead of classifying them with the conventional, gendered way. Although more than 80% of the respondents still chose the gender stereotypic relationship after training, 8.3% of the respondents chose the script relationship. Additionally, the reasons for children’s choice of the script relationship were not limited to the script relationship discussed in the training but included different conceptual systems, such as taxonomic, thematic, and script systems. We speculated that, in script relationship training, the adult demonstrated interpretations of relationships between pictures in a way different from gender stereotyping. This training may have had an implication on the children, prompting them to think about other possible relationships between the pictures. Additionally, children then combined the observed picture clues, their own life experiences, and their multiple conceptual systems to provide a logical explanation of their choices.

When analyzing children’s reasons for their gender stereotypic relationship choice, two subtypes emerged to echo the concepts of stereotype knowledge and stereotype flexibility. The first subtype is the inclination to associate gender with specific behavior, traits, and activity tendency, which shows that children have high gender stereotyping knowledge. The second is the restrictive views of gender and behavioral ability, which shows that children have low gender stereotyping flexibility. As previously suggested, gender stereotypic knowledge is at high levels, and gender stereotype flexibility just begins to show at kindergarten age [43]. 

The GEC intervention was carried out for eight weeks in classroom activities with gender equality picture books, intending to provide gender views different from children’s life experiences, including their observations of parents’ roles in the home. The open questions about the stories provide opportunities for children to share, discuss, and reflect their views. During the intervention, children were especially sensitive to the plots about gender-based bullying or unequal treatment on the protagonists in the picture books, such as *Oliver Button is a Sissy*; *William’s Doll*; *Piggybook*; and *Paper Bag Princess*. When discussing these picture books, children could emphasize the feelings of the protagonists and put forward more flexible views on gender roles and behavior. Especially for the male characters, dresses and activities that were initially attributed to being exclusive to men were loosened. Children gradually accepted the ideas that men can wear long hair, wear makeup, tie bows, play with dolls, and do housework. The proportion of children in the experimental group choosing the gender stereotypic relationship decreased after the GEC intervention. On the contrary, the proportion of children in the control group choosing the gender stereotypic relationship increased to more than 95%. Statistical analysis shows that those who chose the gender stereotypic relationship were mostly children in the control group. That being said, the GEC intervention has a direct influence on breaking children’s gender stereotyping. 

The effect of the GEC intervention can also be seen in the reasons given by children for choosing the gender stereotypic relationship, which is different between the two groups. The answers of the children in the control group show more rigid gender stereotyping. However, children in the experimental group seemed to be implicitly influenced by the picture books, and they needed to emphasize the rationality of their choices through laborious and complex narratives or admitted that these behaviors can be seen in both genders but are just more familiar with a particular gender. While children in the experimental group expressed gender equality views in the discussion process, 75% still chose the gender stereotypic relationship. The dissociation between children’s knowledge of versus the belief in a social stereotype is best explained in dual-process models of social cognition. These models consider that the activation of associative knowledge structures in memory is independent of the propositional validation of activated information [21]. Thus, children may retrieve information discussed in the GEC intervention but do not necessarily regard it as true or accurate. We speculated that this contradiction reflects the difference between picture-book stories and real-life experiences. It can be seen from the children’s answers that they understood gender equality but ultimately still decided to make classification choices based on their observations of people around them in the real-life, especially family members, such as “my daddy does not (clean up)” and “mom usually accompanies me....” It is reasonable for young children to learn the gender role perspectives from their closest family members [44]. Therefore, the promotion of gender equality requires the cooperation of family education.

Overall, the proportion of children in the experimental group choosing the script or other relationships increased to 1/4, reflecting the direct influence of the GEC intervention. Children in the experimental group quoted picture books to explain the reasons for their classification or engaged in more flexible discussions with various expressions, such as “boys can also…” and “girls can also…,” which reflects their changes of gender stereotyping. The remarked effect of the GEC intervention was probably achieved from the prolonged and active engagement in discussion of gender issues, with which children were able to reconstruct their gender cognition. 

### 4.2. Effects of Background Factors

This study further explored the relationship between children’s classification choice and background factors, including children’s gender and age in months. Three types of classification choices were compared, and the classification choice of AA was used for comparison.

Children’s gender was significantly correlated with the three classification choices. The proportion of girls choosing the gender stereotypic relationship was more than 90%, which was higher than that of boys. The finding may be because girls have an understanding of gender earlier than boys [11]; thus, girls have a more stable perception of the gender stereotypic relationship. However, there was no correlation between classification choice AA and gender. In other words, there are no gender differences after the GEC intervention on loosening children’s gender stereotyping. Children, regardless of gender, can benefit from the GEC. 

There were no significant correlations between children’s age in months and their classification choices. These results may be due to the small range in participants’ age and the small sample size. According to a meta-analysis of Signorella et al. [23], children’s gender stereotypic knowledge increased from 3 to 7 years in 45 studies that used a forced-choice male–female answer format. On the other hand, children’s gender stereotype flexibility measured when including a “both” answer category decreased until primary school age before it rebounded at the age of 10 years in 54 studies [23]. Thus, if a more extensive age range and a larger number of children were recruited, the age effect could have been more apparent.

### 4.3. Limitations

The participants in the present study were kindergarten children, and picture pairing was used to facilitate children’s understanding and operation. However, the picture content design was limited to explicit features that were easily visualized, such as toys, professional work, and family affairs. In future research, measurement tools containing implicit aspects, such as personality traits, can be developed to gain a more in-depth and comprehensive understanding of children’s gender stereotyping. We also found that, although the experimental group children were enthusiastically engaged in gender equality conversations, less than one-third of them made different choices after the GEC intervention. The limited impact may be related to the fact that the intervention period lasted not long enough to overturn their gender views learned from their family experiences. Therefore, in addition to prolonging the intervention time, discussions can also be extended from picture book stories to children’s original family situations, which may help young children connect gender equality views with their real family life experience.

## 5. Conclusions

The GEC intervention had a significant effect on loosening children’s gender stereotyping. Not only did the children introspectively assess the appropriateness of a gender stereotype during the discussion after shared reading of picture books but also they chose less gender stereotypic relationship than that of the control group. Even if the children in the experimental group chose gender stereotypic classifications, their reasons were quite different from those in the control group, including taxonomic, thematic, and script systems. Some children used laborious and complicated narratives to emphasize their choice’s appropriateness. Others said both genders could match, but they still chose the pairs in line with the gender stereotype according to their life experiences. 

Our results indicated that, the higher the paternal education level, the stronger the children’s gender stereotypes. We speculated that social desirability tendencies appear to be strongest among people with higher levels of education because of their greater awareness of what are appropriate responses [45]. Fathers had stronger explicit stereotypes than mothers [46]. Whether it is the various disciplines directly given to children by fathers or children develop adaptive behaviors through observing and imitating the characteristics or behaviors of their father, it is reasonable for young children to learn gender role perspectives from their closest family members. Consequently, the promotion of gender equality requires the cooperation of family education.

The current study provides evidence that kindergarten children can learn new gender concepts from various cognitive training even though this age of children is in the most stable period of gender stereotyping. The GEC implemented by script relationship training seems to entice children to rethink about the correlation between pictures from a perspective other than gender stereotyping.

## Figures and Tables

**Table 1:** Content of pictures in the classification task.

Set	Target	Stimulus A	Stimulus B	Script Relationship
1	Long hair	Skirt	Trousers	Window shopping
2	Short hair	Razor	Lipstick	Supermarket
3	Doll	Bow tie	Tie	Tie up
4	Soldier	Toy car	Toy kitchen	Toy shop
5	Boy	Robot	Doll	Toy shop
6	Girl	Broom	Heavy box	Household
7	Little baby	Mother	Father	Family
8	Hammer	Father	Mother	Family

**Table 2:** Participant characteristics.

	Control Group (*n* = 26)	Experimental Group (*n* = 28)	*p* Value
Gender			>0.05
Boy	14 (53.85%)	16(57.14%)	
Girl	12 (46.15%)	12(42.85%)	
Age by months	72.04 (61–78)	72.71 (63–79)	>0.05
Parent education			
Father			>0.05
Above junior college	17 (65.4%)	16 (57.1%)	
Below junior college	9 (34.6%)	12 (42.9%)	
Mother			>0.05
Above junior college	11 (42.3%)	13 (46.4%)	
Below junior college	15 (57.7%)	15 (53.6%)	
Parent job			
Father			>0.05
White-collar professionals	14 (53.8%)	14 (50%)	
Blue-collar workers	12 (46.2%)	14 (50%)	
Mother			>0.05
White-collar professionals	7 (26.9%)	9 (32.1%)	
Blue-collar workers	19 (73.1%)	19 (67.9%)	

**Table 3:** The test of homogeneity for classification choice before intervention.

Variables	Gender Stereotype Counts (%)	Script Relationship Counts (%)	Other Relationships Counts (%)	*χ* ^2^
Girl (*n* = 24)	179 (93.23%)	7 (3.65%)	6 (3.13%)	11.31 **
boy (*n* = 30)	199 (82.92%)	29 (12.08%)	12 (5.00%)	
Total (*n* = 54)	378 (87.50%)	36 (8.33%)	18 (4.17%)	

** *p* < 0.01.

**Table 4:** The test of homogeneity for classification choice after intervention.

Variables	Gender Stereotype Counts (%)	Script Relationship Counts (%)	Other Relationships Counts (%)	*χ* ^2^
Experimental (*n* = 28)	164 (73.21%)	23 (10.27%)	37 (16.52%)	45.67 ***
Control (*n* = 26)	200 (96.15%)	7 (3.37%)	1 (0.48%)	
Total (*n* = 54)	364 (84.25%)	30 (6.94%)	38 (8.80%)	

*** *p* < 0.001.

## Data Availability

Data sharing is not applicable for this article.
